# Real-world effectiveness of COVID-19 vaccines among Colombian adults: A retrospective, population-based study of the ESPERANZA cohort

**DOI:** 10.1371/journal.pgph.0001845

**Published:** 2023-09-08

**Authors:** Maylen Liseth Rojas-Botero, Julián Alfredo Fernández-Niño, Leonardo Arregocés-Castillo, Andrés Palacios-Clavijo, Mariana Pinto-Álvarez, Fernando Ruiz-Gómez

**Affiliations:** 1 Facultad Nacional de Salud Pública, Universidad de Antioquia, Medellín, Colombia; 2 Johns Hopkins Bloomberg School of Public Health, Baltimore, Maryland, United States of America; 3 Universidad del Norte, Barranquilla, Colombia; 4 Pontificia Universidad Javeriana, Bogotá, Colombia; 5 Independent Researcher, Bogotá, Colombia; 6 Fundación Universitaria Sanitas, Bogotá, Colombia; 7 Processum SAS, Bogotá, Colombia; Getulio Vargas Foundation, BRAZIL

## Abstract

COVID-19 vaccines have been essential for reducing the impact of the pandemic; nevertheless, population-based data under real-life conditions are needed to compare their effectiveness in various contexts. The objective of this study was to estimate the effectiveness of vaccines in preventing hospitalization and death from COVID-19 in Colombia under real-life conditions among people aged 18 years and older, according to sex, age, confirmed history of COVID-19 and vaccination series, including the effects of boosters. This investigation was an observational, retrospective, population-based study based on the Colombian cohort “Esperanza”. A total of 14,213,409 individuals aged 18 years and older were analyzed, who were matched in a 1:1 ratio of vaccinated to unvaccinated. The study groups consisted of unvaccinated individuals, those with a complete series (CS) and individuals with a CS plus booster. The vaccinated individuals received either homologous or heterologous vaccinations with Ad26.COV2-S, BNT162b2, ChAdOx1 nCoV-19, CoronaVac and mRNA-1273 vaccines. Follow-up was conducted between February 2021 and June 2022. Cox proportional hazards models were used, adjusted for potential confounders, to estimate the effectiveness of different vaccination series. For adults aged 18 years and older, the overall effectiveness of the vaccines in preventing hospitalization was 82.7% (95% CI 82.1–83.2) for CS and 80.2% (95%CI 78.7–81.6) for CS + booster. The effectiveness in preventing death was 86.0% (95%CI 85.5–86.5) for CS and 83.1% (95%CI 81.5–84.5) for CS + booster. Effectiveness decreased with age. While all efficacies were high, CoronaVac offered significantly lower protection, although this improved with a booster. Continued mass vaccination is pivotal, especially in low- and middle-income countries. The study highlights both the real-world effectiveness of these vaccines and the challenges in understanding waning immunity and the influence of different VoC(Variants of Concern) on results.

## Introduction

The COVID-19 pandemic has posed one of the greatest challenges for the world in recent history, having a large impact on morbidity and mortality [1–3]. The challenge of controlling the pandemic has led to an unprecedented collective worldwide effort by the scientific community, the pharmaceutical industry, non-governmental organizations and governments to develop, produce and distribute highly effective vaccines to prevent symptomatic infection [4–8]. Although we now know that their efficacy in reducing transmission was lower than initially estimated, and that transmission decreases over time [9] or is substantially reduced by the emergence of new variants [5, 10], their efficacy in reducing the most severe cases of the disease does appear to continue to be high [11]. Today, roughly two years after national vaccination plans began in most countries, several studies have shown that the global burden of the disease has been substantially reduced thanks to vaccination [12], although this impact would have been much greater and quicker if there had been an equitable global distribution of the vaccines [13, 14]. To generate more robust estimates of deaths prevented worldwide, we need to know the specific effectiveness of vaccines against COVID-19, and their various combinations, in preventing severe forms of disease and death in uncontrolled, real-life settings.

Several studies, some of them at the national level, have shown evidence that vaccination with a complete series is highly effective in preventing symptomatic infection, severe disease, hospitalization and/or death [15]. However, evidence of the effectiveness of boosters in uncontrolled conditions in low and middle-income countries(LMICs) is still limited, especially since they have been administered more recently, and also because many countries, such as Colombia, have used different platforms and subsequent homologous and heterologous boosters, which turned out to be highly diverse configurations and very worthwhile.

Colombia began to implement its National Vaccination Plan (NVP) on February 17, 2021, offering to all inhabitants in the country a broad portfolio of vaccines free of charge, namely: two RNA (mRNA) vaccines, BNT162b2 and mRNA-1273; two adenoviral vector vaccines, ChAdOx1 nCoV-19 and Ad26.COV2.S; and the CoronaVac inactivated virus vaccine. In addition, the Esperanza cohort was established as part of the NVP, which included all inhabitants in Colombia, for a total of 51.6 million people in the year 2022 according to the 2018 national census projections [16]. The present study is nested in this cohort.

Due to the initial shortage of vaccines, the population over 60 years of age was first in the prioritization process for access to vaccines since they were more vulnerable to the virus. Therefore, the analysis of the effectiveness of a complete vaccine series in this population subgroup was previously published [17]. However, with nearly 88 million doses administered in Colombia as of August 2022—for a coverage of a complete series of 70.7%—and with more than 13.7 million booster doses having been administered [18], we need to know the degree to which the vaccines are effective in preventing hospitalization and death among the adult population 18 years of age and older. In addition, Colombia is an ideal context for evaluating effectiveness under real-world conditions because, as in other low- and middle-income countries, it has been using a diversified platform of vaccines. We also consider our results derived from a population-based cohort are valuable for decision-making in others LMICs.

## Materials and methods

### Study design and population

An analytical, observational, population-based cohort study was conducted that matched vaccinated and unvaccinated populations. The purpose was to evaluate the effectiveness of the complete series (equivalent to two doses of all vaccines except Ad26.COV2.S - one dose of Ad26.COV2-S was considered as a completed scheme-) and a booster (homologous or heterologous) in preventing hospitalization and death from COVID-19 among adults aged 18 years and older in Colombia, for all ages and for specific age groups (18 to 44, 45 to 59, 60 to 69, 70 to 79 and 80 years and older).

The “Esperanza" cohort, which means "hope" in Spanish, was established as part of Colombia’s NVP. It included all inhabitants in Colombian who were eligible to receive the COVID-19 vaccine [19]. This study analyzed individuals aged 18 years and older, projected by the 2018 census to be 37.4 million for the year 2022 [16]. Individuals were divided into cohorts according to vaccination series: a closed cohort for the unvaccinated group (comparison group) and dynamic cohorts for individuals with a complete series (CS) and CS + booster. Individuals with partial vaccinations were not included in this analysis (who did not complete the complete vaccination scheme during the study period).

### Information sources

The information on the individuals was obtained from several databases using an anonymized individual identification code, using the same procedure described in the evaluation of the effectiveness of vaccines in the older adult population in Colombia [17]. These sources are integral components of the integrated social protection information system, SISPRO, which streamlined the cross-referencing and validation of data from multiple sources while adhering to the data quality standards established by this framework. The databases incorporated in the study encompassed:

- Mi Vacuna, which contains socio-demographic data for all people eligible to receive the COVID-19 vaccine in Colombia (sex, age—calculated using the date of birth- and municipality of residence);
- PAIWEB, which registers the vaccines that are administered at the individual level;
- SEGCOVID, which provides follow-up data on confirmed COVID-19 cases, including hospitalization;
- RUAF-ND, which contains records of deaths from all causes, including those due to COVID-19.
- SISMUESTRAS, which includes all PCR and antigen tests, based on which individuals with a confirmed history of COVID-19 are identified;
- BDUA, where enrollment in the Colombian health system can be found; and
- High Cost Account, which is a registry of patients in the health system who have high-cost diseases and identifies those who have been diagnosed with chronic kidney disease, hypertension, diabetes and cancer.

Notably, "Mi Vacuna" is a database constructed based on information related to access to social security, utilizing the Unique Affiliates Database (BDUA, in Spanish: Base única de afiliados). This database is extensively used in Colombia for cohort construction and demonstrates strong consistency with census data, given the high coverage of the social security system, exceeding 99% for Colombian citizens and regular residents.

Unfortunately, access to the Civil Registry information was not available to us as it is under the custodianship of the National Statistics Directorate (DANE) and cannot be shared with external entities. The process of cross-referencing and data integration was conducted by specialized professionals in the Office of Technology and Information Sources, ensuring strict adherence to data confidentiality and privacy protocols. As researchers, we were granted access solely to anonymized data upon completion of the integration process.

S1 Fig depicts a flowchart outlining the preparation and integration of the databases utilized in this analysis.

### Selection criteria

This study included all adults aged 18 years and older who did not receive any dose of a COVID-19 vaccine during the observation period and those who completed a vaccination series, some of whom later received a booster dose, and some did not. It excluded people who had no sex recorded, incomplete information about vaccination series (missing information about prior doses) and partial vaccinations, as well as records that had quality problems on the date of the events of interest (vaccination dates that were prior to the start of the NVP, vaccination dates after the cut-off date of the analysis, dates of occurrence of events or administration of doses after the date of death was registered).

### Procedure for generating the cohort

After applying the selection criteria to the Esperanza cohort, 28,539,635 individuals were identified as being eligible for this study, of which 6,498,915 were unvaccinated, 22,040,720 had a CS and 6,193,557 of those also received at least one booster.

Two independent iterative matching processes were conducted: one to analyze the effectiveness of the CS and one for the effectiveness of CS + booster. Based on this, an observation start date was assigned to individuals in the unvaccinated cohort in each analysis, where each of these individuals had the probability of being observed over time. To this end:

1. Individuals were grouped who had the same characteristics in terms of sex, age (in single ages) and health system enrollment (contributory or subsidized).
2. In each group, the individuals were randomly ordered (using a uniform distribution), first those exposed to vaccination and then those not exposed. A consecutive number was assigned to each individual.
3. The vaccinated individual with the lowest consecutive number was provisionally paired with the non-vaccinated individual with the lowest consecutive.
4. The follow-up start date for the vaccinated individuals was the 15th day after the administration of the vaccine. This same calendar date was assigned to the provisionally matched unvaccinated individual. Unvaccinated individuals were those who did not receive any vaccine (not even a dose) during the entire study period.
5. If the unvaccinated individual had an event of interest, such as first hospitalization or death from COVID-19, or had died from any cause prior to the vaccinated individual’s follow-up start date, then the matching was undone. The unvaccinated individual was then moved to the bottom of the unexposed group list and the exposed individual was paired with the next unvaccinated individual in the ordered list. If no event occurred, then the pairing was confirmed.
6. This process was performed iteratively and ended when there were no individuals left to pair or when all pairs had valid entry dates. Unpaired individuals were eliminated from the analysis.

It should be noted that the same individual could have been selected for the CS analysis and for the CS + booster analysis. The pairing process resulted in 5,709,210 pairs for the analysis of the effectiveness of the CS and 3,901,818 pairs for the analysis of the CS + booster. July 5, 2022 was the last date for querying the databases.

Individuals with a CS who subsequently received a booster were censored from the CS analysis. In this case, these individuals were followed as of day 15 after completing the vaccination series and until day 14 after receiving the booster. The same was done with the CS + booster analysis when the individual received a second booster.

The matching was fixed for all subjects; no replacement or reassignment of the matches was conducted.

### Follow-up period

The observation period was 489 days, from February 17, 2021 to June 20, 2022, during which each individual was followed for a particular amount of time. For each vaccinated CS-unvaccinated pair, follow-up began 15 days after the vaccinated individual in the pair received the dose that completed the series (the time required for the vaccine to produce an immune response) and culminated on the day of occurrence of the event (hospitalization or death from COVID-19) or censoring. Similarly, the follow-up time for each CS + booster-unvaccinated pair began on day 15 after the vaccinated individual received the booster dose and culminated with the event or censoring.

There were three types of right censoring: individuals who died from causes other than COVID-19, those who received the booster (first booster with the CS and second booster with the CS + booster) and those who completed the follow-up and observation period without having presented the outcome of interest. In both analyses, the unvaccinated individual was also censored when their counterpart received the booster, in order to control for the observation period and the circulating variants.

### Outcomes

The effectiveness of vaccines in preventing hospitalization and death from COVID-19 was analyzed based on the definitions recommended by the World Health Organization [20]. Death from COVID-19 was defined as death resulting from a clinically compatible disease in a probable or confirmed case of COVID-19, unless there was a clear alternative cause of death that could not be related to COVID-19, with no defined complete recovery period between illness and death [21].

Analysis of overall effectiveness was also stratified by COVID-19 history. History of COVID-19 was defined as having had another confirmed COVID-19 infection before first hospitalization for COVID-19 and (obviously) before dying from COVID-19, this includes another confirmed infection that occurred within the period of study (or even before the study began) but that occurred before death or any hospitalization from COVID-19. To ensure the differentiation of distinct infections, we implemented a time-based criterion. Specifically, if two laboratory-confirmed SARS-CoV-2 infections were reported within one month, they were considered as likely being the same infection and not counted as separate events. For subjects who experienced hospitalization or death, we considered a prior confirmed infection as distinct from the outcomes of interest if it occurred more than one month before the onset of any complication related to COVID-19 that resulted in hospitalization or death during the follow-up period.

### Statistical analysis

Cox proportional hazards models were used for the survival analysis, adjusted for the following possible confounders: sex; age; health system enrollment; diagnosis of cancer, diabetes, hypertension or chronic kidney disease; confirmed history of COVID-19; and municipality of residence. The selection of covariates was based on a directed acyclic graph (DAG) presented in S3 Fig. This DAG guided the identification of relevant variables to include in the analysis.

Vaccine effectiveness was calculated as (1-HR) ∙100% for both the population aged 18 years and older and for each specific age group: 18 to 44 years, 45 to 59 years, 60 to 69 years, 70 to 79 years and 80 years and older.

Assumptions about proportionality of risk over time were evaluated for each model based on the behavior of [-log〖 (*probability of survival*) 〗] with respect to log〖 (*time*)〗 for each event of interest. The curves were parallel in all cases (S2 Fig).

The results are presented with texts and figures. The analysis was conducted with the R (version 4.2.0) survival package version 3.3–1 to estimate Kaplan-Meier functions and fit Cox proportional hazards regression models. The data and results were visualized using ggplot2 (version 3.3.6)

### Sensitivity analysis

A first sensitivity analysis was performed to explore whether the censorship of vaccinated patients who subsequently received the booster. The analysis was performed without censoring unvaccinated individuals when their counterpart received the booster, both for the analysis of CS and CS + booster. Effectiveness did not show significant differences, and the results were consistent. An additional sensitivity analysis tried to explore the potential effect of changes in the predominant variants in Colombia on the effectiveness in each age group, for which the effectiveness by age group was estimated in two different time periods. The first, from February 2021 to November 2021, corresponding to the period before the appearance of the Omicron variant and in which Mu predominated in Colombia, versus the period from December 2021 to June 2022 in which Omicron predominated. The periods with the predominant variant between 2021 and 2022 in Colombia are presented in S1 Table.

### Ethics statement

This investigation meets the scientific, technical, administrative and ethical considerations stipulated by existing regulations for research with human beings in Colombia. In accordance with 1993 resolution 8430, this study is classified as no risk since it exclusively uses secondary data sources. None of the study researchers accessed the databases that contained the original personal identifiers, they used only anonymized databases. All standards for handling information were followed. Given these factors, this study did not require review or approval by a research ethics committee.

The Ministry of Health and Social Protection is regulated by national legislation on information management, habeas data laws, and institutional manuals on best practices. All information sources were directly managed by the Ministry of Health, and the databases were anonymized, joined and made available for use by this study by independent technicians who linked the sources using their own encrypted key code, without using the original personal citizen identification. Therefore, it was not possible for researchers or external agents to recover the original identity numbers or personal data.

## Results

### Characterization of the study population

A total of 5,709,210 exposed-unexposed pairs were included in the analysis of the effectiveness of the CS and 3,901,818 pairs were included in the analysis of the CS + booster. Table 1 summarizes the social, demographic and clinical characteristics as well as the COVID-19 vaccination series.

**Table 1:** Social, demographic and clinical characteristics of the population, according to study group.

	Unvaccinated	Vaccinated	Total
*Effective analysis for the CS*	(n = 5,709,210)	(n = 5,709,210)	(n = 11,418,420)
**Sex (%)**			
Men	50.5	50.5	50.5
**Age**			
Mean (SD)	49.2 (16.6)	49.2 (16.6)	49.2 (16.6)
Median (Q1; Q3)	48.0 (40.0; 59.0)	48.0 (40.0; 59.0)	48.0 (40.0; 59.0)
**Age group (%)**			
18–44	39.9	39.9	39.9
45–59	35.7	35.7	35.7
60–69	13.0	13.0	13.0
70–79	6.5	6.5	6.5
80 and over	4.9	4.9	4.9
**Health system enrollment (%)**			
Contributory	41.1	41.1	41.1
Subsidized	58.9	58.9	58.9
**Comorbidities (%)**			
Cancer	0.5	0.8	0.7
Diabetes	2.5	4.3	3.4
Hypertension	8.2	13.8	11.0
Chronic kidney failure	1.3	2.4	1.8
At least one comorbidity	9.7	15.8	12.7
**History of confirmed COVID-19 (%)**	**10.7**	**9.8**	**10.3**
**Vaccination series n (%)**			
***Homologous***	**-**	**4787900(83.9)**	**4787900**
Ad26.COV2-S	-	1261243(22.1)	1261243
BNT162b2	-	1110270(19.4)	1110270
ChAdOx1 nCoV-19	-	672388 (11.8)	672388
CoronaVac	-	1244107 (21.8)	1244107
mRNA-1273	-	499892 (8.8)	499892
***Heterologous***	**-**	**921310 (16.1)**	**921310**
ChAdOx1 nCoV-19+ BNT162b2	-	11377 (0.2)	11377
ChAdOx1 nCoV-19+ mRNA-1273	-	4440 (0.1)	4440
mRNA-1273+ BNT162b2	-	5284 (0.1)	5284
Other combinations	-	900209 (15.8)	900209
***Effectiveness analysis for the CS + booster***	**(n = 3901818)**	**(n = 3901818)**	**(n = 7803636)**
**Sex (%)**			
Men	54.1	54.1	54.1
**Age**			
Mean (SD)	50.6 (17.1)	50.6 (17.1)	50.6 (17.1)
Median (Q1; Q3)	50.0 (39.0; 63.0)	50.0 (39.0; 63.0)	50.0 (39.0; 63.0)
**Age group (%)**			
18–44	38.0	38.0	38.0
45–59	31.3	31.3	31.3
60–69	16.2	16.2	16.2
70–79	9.1	9.1	9.1
80 and older	5.3	5.3	5.3
**Health system enrollment (%)**			
Contributory	56.8	56.8	56.8
Subsidized	43.2	43.2	43.2
**Comorbidities (%)**			
Cancer	0.6	1.1	0.9
Diabetes	2.9	5.4	4.2
Hypertension	9.7	17.5	13.6
Chronic kidney failure	1.6	3.2	2.4
At least one comorbidity	11.3	20.0	15.7
**History of confirmed COVID-19 (%)**	16.0	15.5	15.7
**Vaccination series n (%)**			
***Homologous***	-	**1555073 (39.9)**	**1555073**
Ad26.COV2-S	-	402497 (10.3)	402497
BNT162b2	-	510276 (13.1)	510276
ChAdOx1 nCoV-19	-	171767 (4.4)	171767
CoronaVac	-	411433 (10.5)	411433
mRNA-1273	-	59100 (1.5)	59100
***Heterologous***	-	**2346745 (60.1)**	**2346745**
BNT162b2+ BNT162b2+ ChAdOx1 nCoV-19	-	347060 (8.9)	347060
BNT162b2+ BNT162b2+ mRNA-1273	-	385656 (9.9)	385656
CoronaVac + CoronaVac + BNT162b2	-	319176 (8.2)	319176
CoronaVac + CoronaVac + ChAdOx1 nCoV-19	-	260217 (6.7)	260217
CoronaVac + CoronaVac + mRNA-1273	-	285738 (7.3)	285738
Other combinations	-	748898 (19.2)	748898

· Not applicable

As a result of the matching process, the distribution of sex, age, and health system enrollment status was the same for vaccinated and unvaccinated cohorts. Vaccinated cohorts tended to have a higher proportion of comorbidities and the unvaccinated cohorts had a slightly higher proportion of confirmed history of COVID-19.

For each analysis, the median follow-up time was the same for vaccinated and unvaccinated cohorts (Table 2). During this period there were a total of 25,674 hospitalizations and 19,835 deaths, most of which occurred among adults over 70 years and the unvaccinated cohorts.

**Table 2:** Occurrence of the main study outcomes and mean follow-up time according to cohort and effectiveness analysis.

	Complete Series(CS) analysis			Complete Series + booster analysis		
	Unvaccinated	CS vaccination	Total	Un-vaccinated	CS + booster	Total
*Outcome (n)*						
Hospitalization	16485	5562	22047	2396	1231	3627
Death	13416	3840	17256	1820	759	2579
*Follow-up period*						
Median, days (Q_25_ –Q_75_)	248 (195 – 326)	248 (195 – 326)	248 (195 – 326)	126 (96 – 167)	126 (96 – 167)	126 (96 – 167)

**Fig 1:**
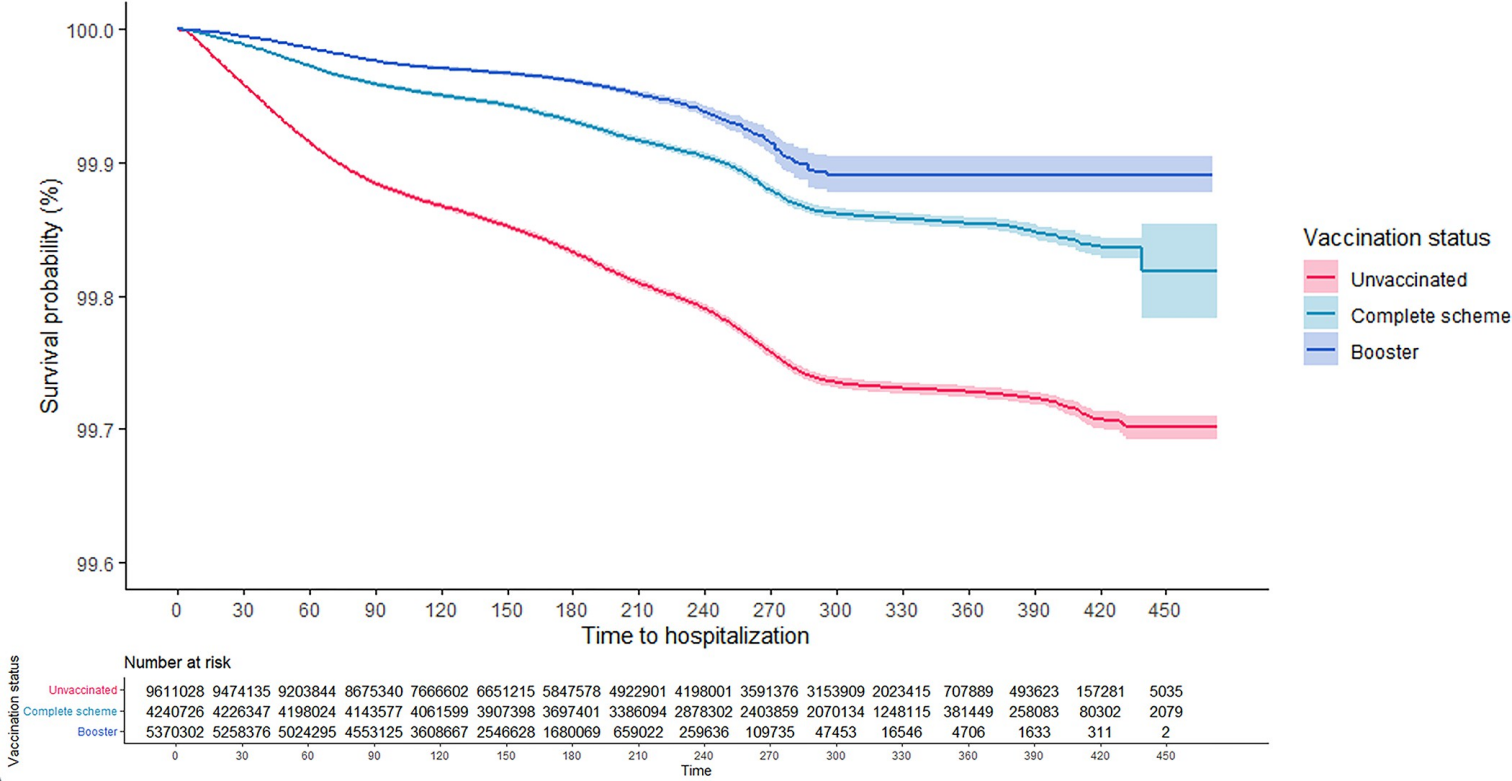
Kaplan-Meier survival curves for hospitalization due to COVID-19 among adults aged 18 years and older in Colombia by vaccination status: Overall.

**Fig 2:**
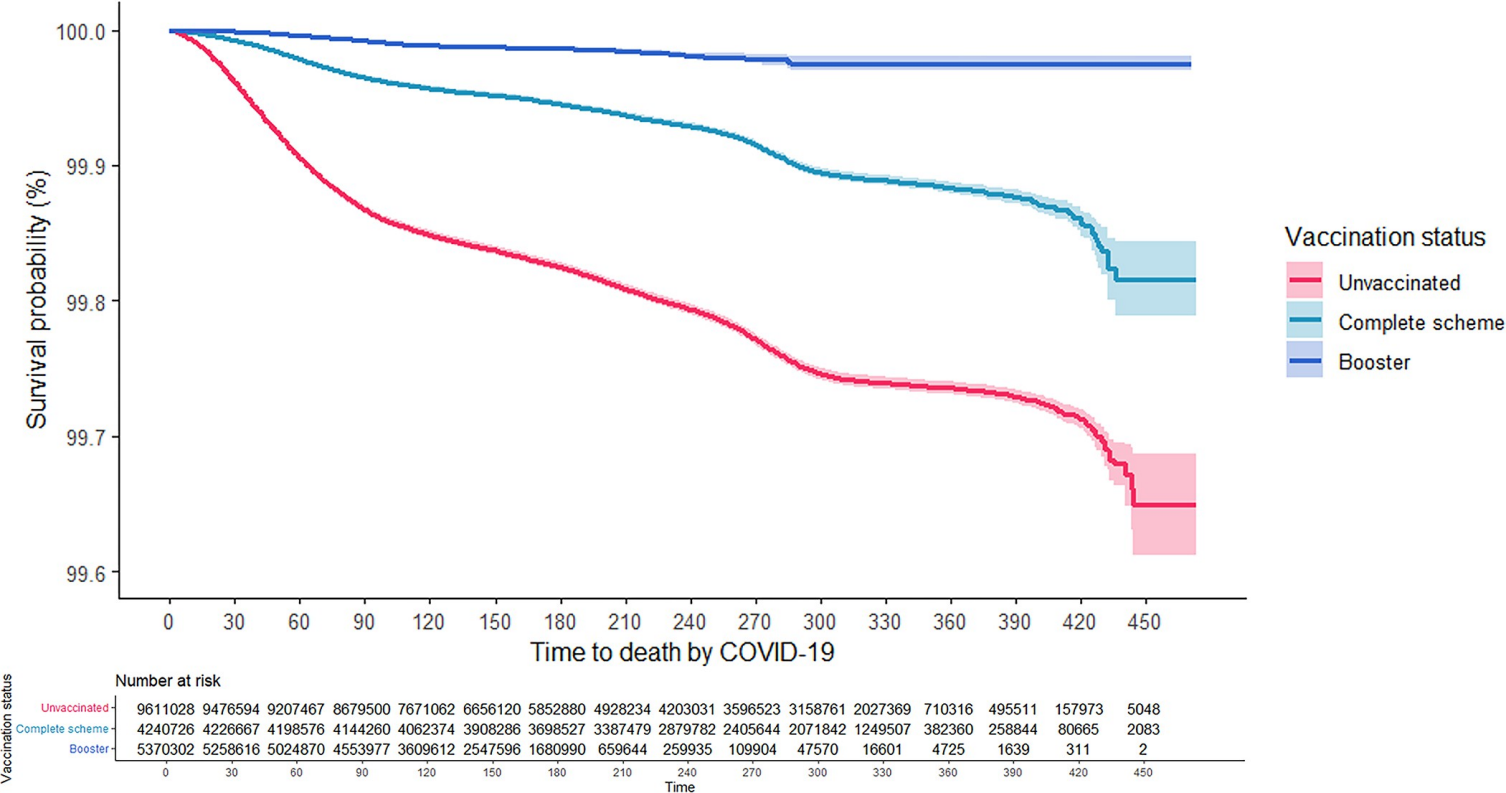
Kaplan-Meier survival curves for death due to COVID-19 among adults aged 18 years and older in Colombia by vaccination status: Overall.

**Fig 3:**
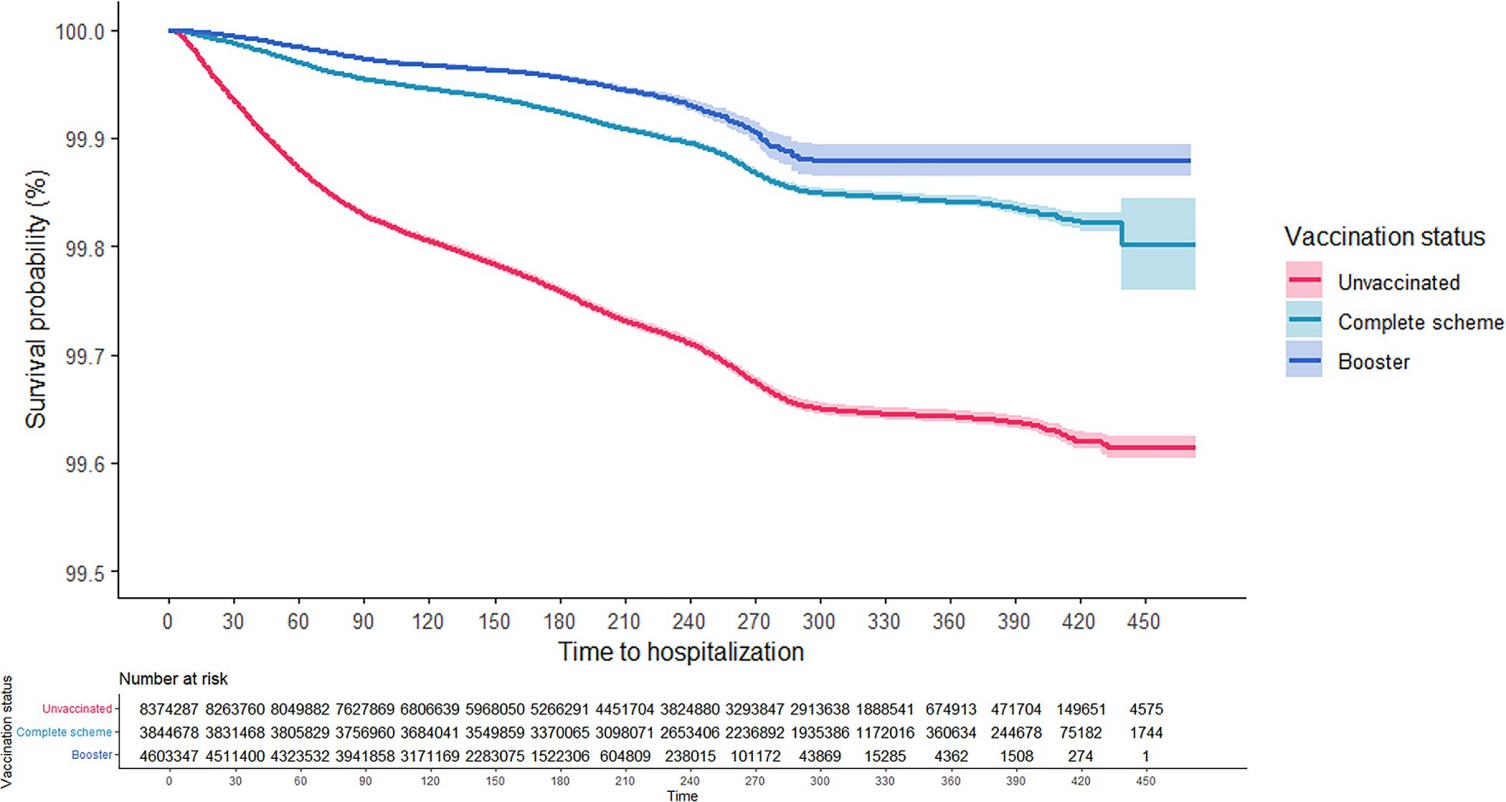
Kaplan-Meier survival curves for hospitalization due to COVID-19 among adults aged 18 years and older in Colombia by vaccination status: People without a previous confirmed history of COVID-19.

**Fig 4:**
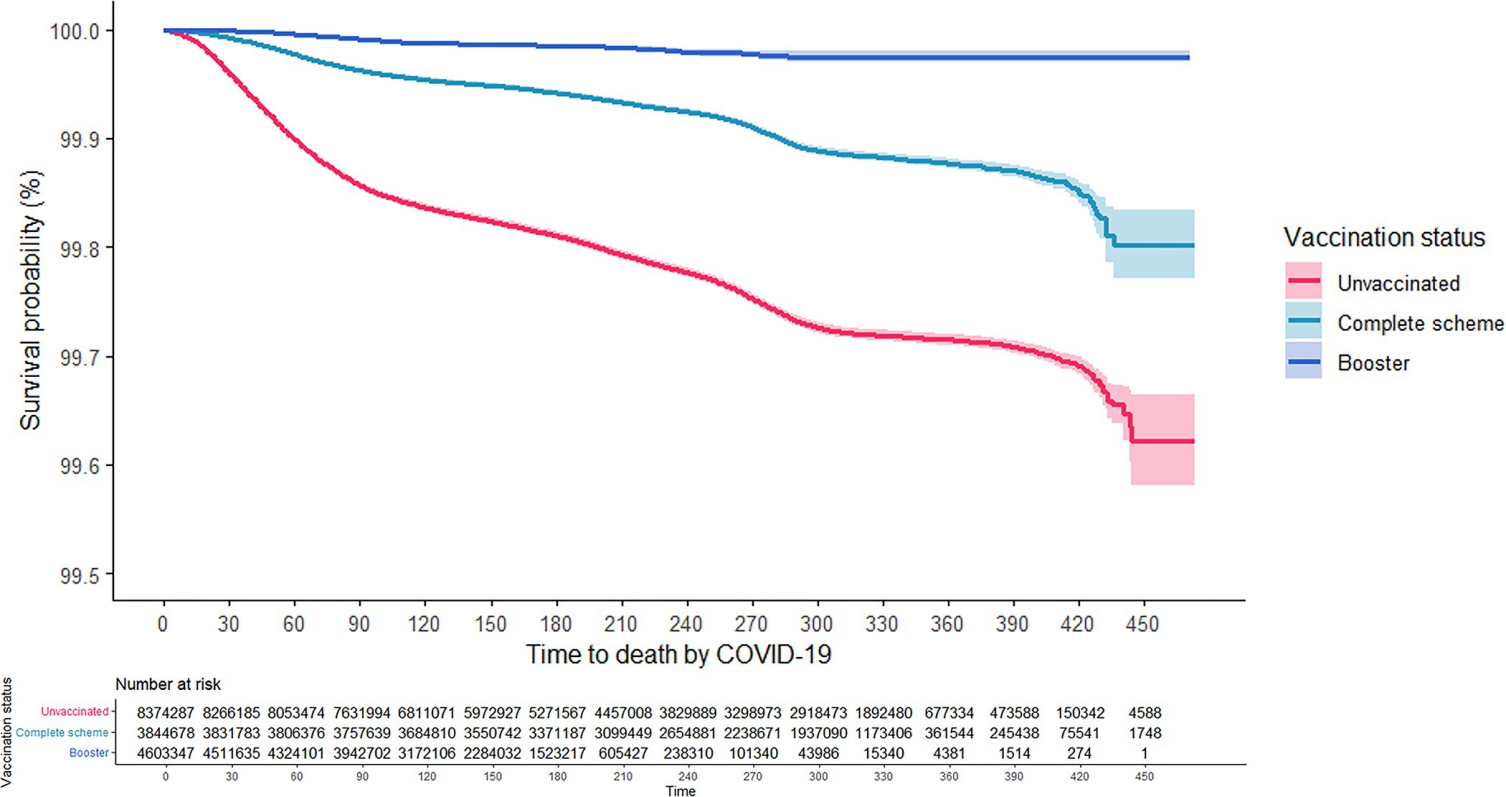
Kaplan-Meier survival curves for death due to COVID-19 among adults aged 18 years and older in Colombia by vaccination status: People without a previous confirmed history of COVID-19.

**Fig 5:**
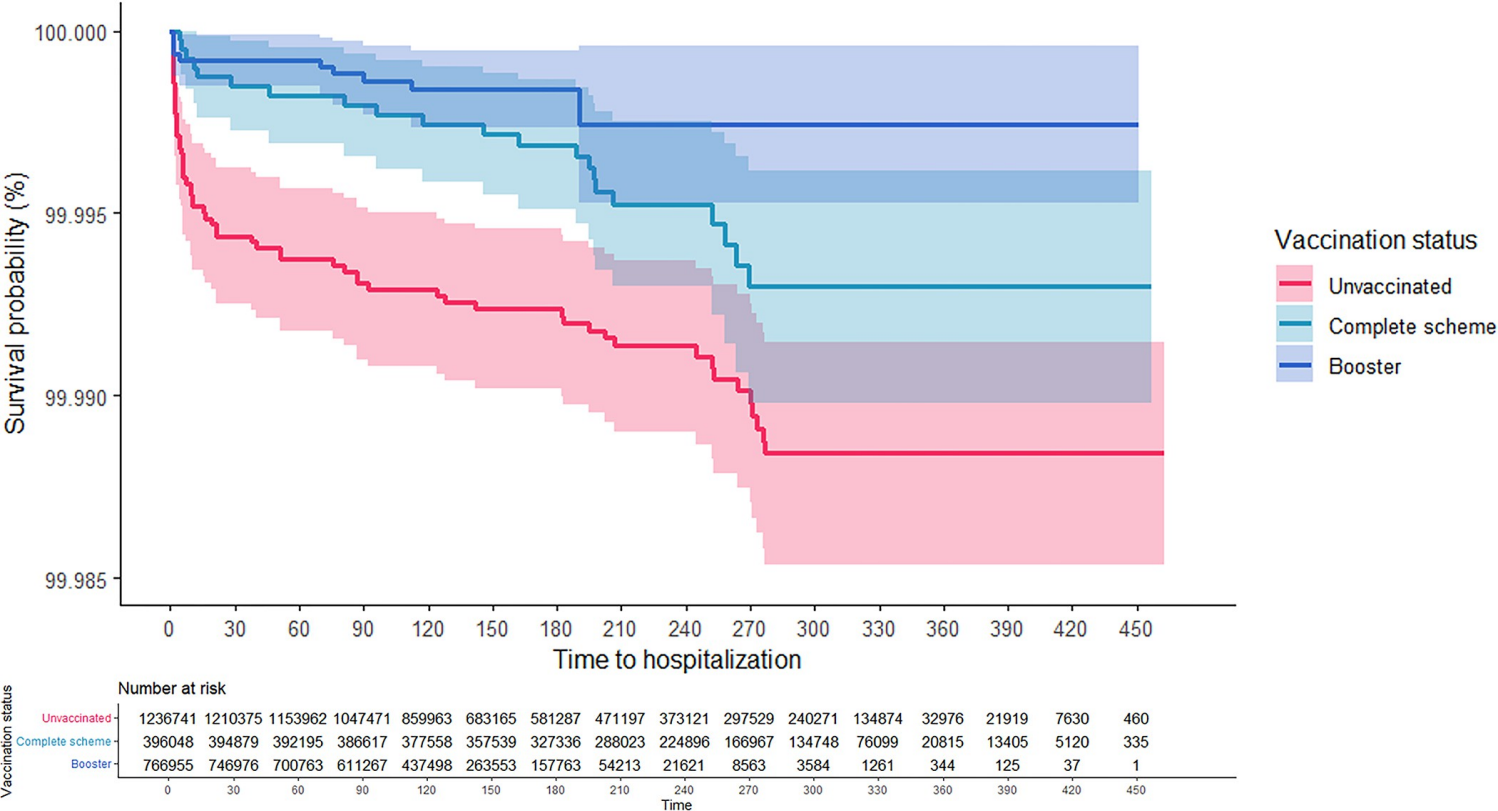
Kaplan-Meier survival curves for hospitalization due to COVID-19 among adults aged 18 years and older in Colombia by vaccination status: People with a previous confirmed history of COVID-19.

**Fig 6:**
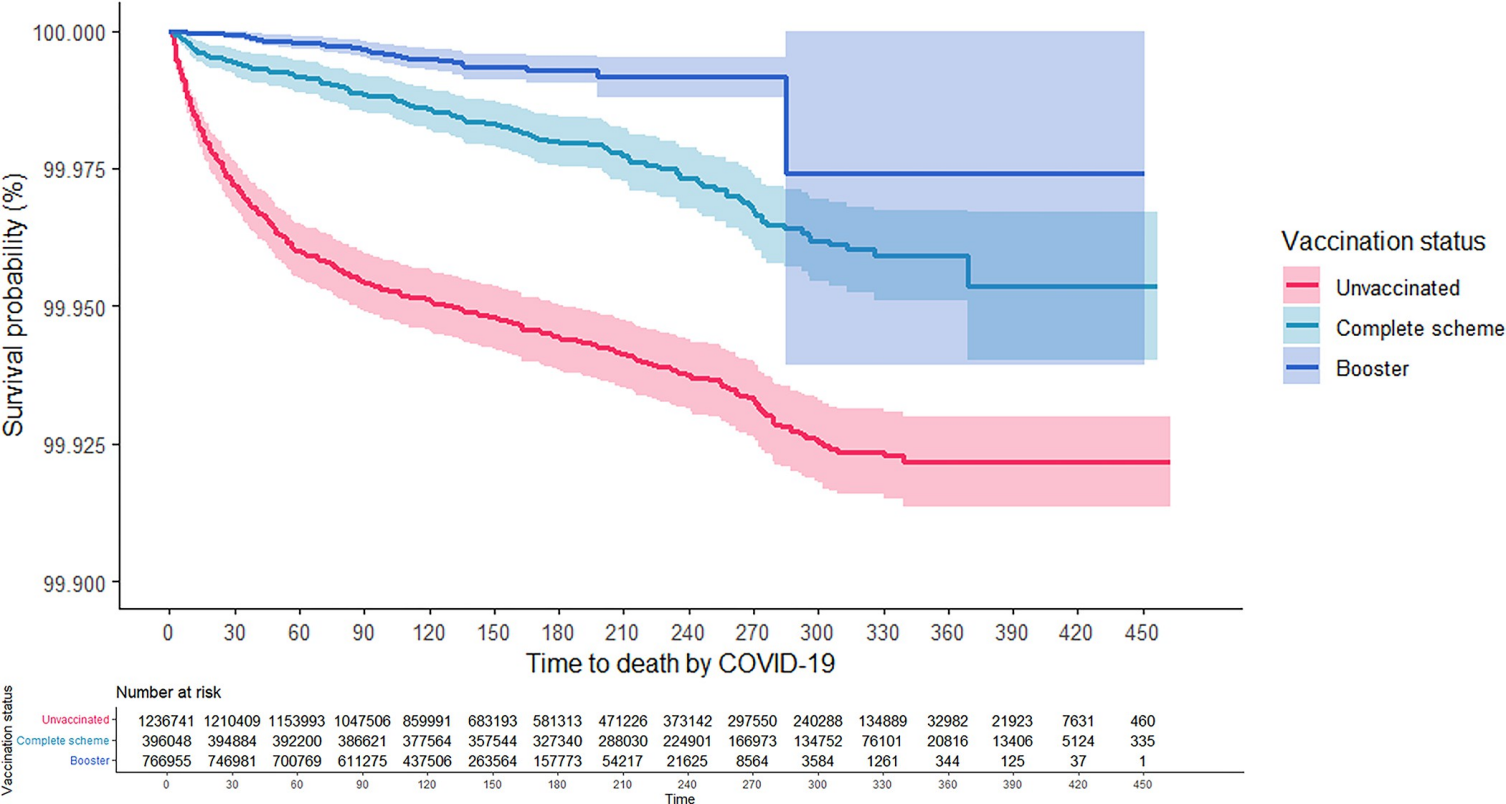
Kaplan-Meier survival curves for death due to COVID-19 among adults aged 18 years and older in Colombia by vaccination status: People with a previous confirmed history of COVID-19.

As can be seen in the Kaplan-Meier survival curves, the risk of hospitalization and death from COVID-19 for adults aged 18 years and older was consistently higher among the unvaccinated, while those with CS + booster had the lowest risk (Figs 1 and 2). These results were consistent in the analysis that was stratified according to confirmed history of COVID-19 (Figs 3–6). In all the Figures for vaccinated people, time 0 corresponds to 15 after finishing the scheme, as explained in methods.

### Effectiveness in preventing hospitalization

For adults who were vaccinated with a CS, the adjusted vaccine effectiveness (aVE) in preventing hospitalization from COVID-19 was 82.7% (95%CI 82.1–83.2). This effectiveness was significantly less in people aged 80 years and older (75.2%, 95%CI 74.0–76.3). The aVE for a CS was highest for those who received two doses of mRNA-1273 (93.7%, 95%CI 91.8–95.2) and lowest for those who received two doses of CoronaVac (77.4% 95%CI 76.5–78.2), with statistical differences (Table 3).

**Table 3:** Effectiveness of COVID-19 vaccines in preventing hospitalization and death among adults aged 18 years and older by age group, vaccination series and vaccine.

		Complete Series (95% CI)		Complete Series + booster (95% CI)	
		Hospitalization	Death	Hospitalization	Death
Age group					
	**18 years and older**	**82.7% (82.1 – 83.2)**	**86.0% (85.5 – 86.5)**	**80.2% (78.7–81.6)**	**83.1% (81.5–84.5)**
	18–44 years	91.5% (90.4 – 92.4)	95.1% (93.5 – 96.3)	89.7% (85.1–92.9)	89.5% (74.0–95.8)
	45–59 years	83.2% (81.6 – 84.7)	90.5% (89.0 – 91.8)	71.5% (62.4–78.4)	74.0% (59.5–83.3)
	60–69 years	87.1% (86.0 – 88.2)	91.7% (90.7 – 92.6)	81.3% (77.0–84.8)	83.8% (78.4–87.9)
	70–79 years	85.9% (84.9 – 86.8)	90.0% (89.1 – 90.8)	84.0% (81.5–86.1)	86.8% (84.2–89.0)
	80 years and older	75.2% (74.0 – 76.3)	78.5% (77.4 – 79.5)	76.8% (74.3–79.1)	80.3% (77.9–82.5)
Vaccine: **Ad26.COV2-S**		One dose		Two doses	
	18 years and older	85.4% (83.8 – 86.9)	90.5% (88.8 – 91.9)	89.0% (78.7–94.3)	77.6% (55.0–88.9)
	18–44 years	90.7% (88.9 – 92.2)	93.1% (89.7 – 95.4)	95.6% (86.0–98.6)	--
	45–59 years	78.0% (73.6 – 81.7)	86.0% (81.1 – 89.6)	71.9% (11.0–91.1)	--
	60–69 years	80.6% (74.5 – 85.2)	88.0% (82.3 – 91.8)	--	--
	70–79 years	82.7% (76.4 – 87.4)	88.2% (82.5 – 92.1)	87.1% (83.0–98.2)	--
	80 years and older	86.4% (80.0 – 90.7)	86.9% (80.9 – 91.1)	--	--
Vaccine: **BNT162b2**		Two doses		Three doses	
	18 years and older	90.5% (89.8 – 91.2)	93.5% (92.8 – 94.2)	89.6% (86.0–92.2)	89.6% (84.5–92.9)
	18–44 years	91.2% (89.1 – 92.9)	97.0% (93.7 – 98.6)	87.0% (71.7–94.0)	--
	45–59 years	87.7% (85.8 – 89.3)	93.8% (92.0 – 95.1)	82.8% (67.0–91.0)	82.3% (50.9–93.6)
	60–69 years	90.7% (89.3 – 91.9)	93.1% (91.7 – 94.3)	86.7% (78.1–91.9)	88.0% (75.3–94.1)
	70–79 years	92.4% (91.0 – 93.6)	94.9% (93.7 – 95.9)	93.9% (88.3–96.9)	91.6% (83.0–95.8)
	80 years and older	88.5% (85.4 – 90.9)	89.1% (86.2 – 91.4)	88.8% (70.1–95.8)	90.2% (69.5–96.9)
Vaccine: **ChAdOx1 nCoV-19**		Two doses		Three doses	
	18 years and older	88.0% (86.8 – 89.1)	92.6% (91.5 – 93.6)	78.4% (72.6–82.9)	84.5% (77.9–89.2)
	18–44 years	97.3% (95.4 – 98.4)	99.1% (93.4 – 99.9)	--	--
	45–59 years	85.5% (81.0 – 89.0)	91.4% (86.5 – 94.5)	87.0% (69.0–98.2)	--
	60–69 years	88.1% (86.0 – 89.9)	94.2% (92.5 – 95.6)	71.2% (58.6–80.0)	80.5% (64.8–89.2)
	70–79 years	90.5% (88.7 – 91.9)	94.3% (92.9 – 95.4)	82.4% (74.8–87.6)	87.1% (78.6–92.2)
	80 years and older	81.7% (76.9 – 85.5)	87.9% (84.0 – 90.9)	85.2% (60.6–94.5)	86.8% (58.8–95.7)
Vaccine: **CoronaVac**		Two doses		Three doses	
	18 years and older	77.4% (76.5 – 78.2)	81.9% (81.2 – 82.7)	71.3% (68.3–74.0)	76.3% (73.3–79.0)
	18–44 years	87.5% (84.6 – 89.8)	92.7% (88.0 – 95.6)	90.4% (69.2–97.0)	--
	45–59 years	76.3% (72.8 – 79.4)	86.8% (83.3 – 89.5)	62.0% (31.1–79.1)	--
	60–69 years	82.6% (80.2 – 84.6)	88.1% (85.9 – 90.0)	80.1% (67.6–87.8)	64.9% (38.5–79.9)
	70–79 years	81.7% (80.2 – 83.1)	86.6% (85.3 – 87.7)	75.5% (70.2–79.8)	81.2% (75.7–85.5)
	80 years and older	73.6% (72.2 – 74.8)	77.0% (75.8 – 78.1)	68.4% (64.2–72.1)	74.9% (71.0–78.2)
Vaccine: **mRNA-1273**		Two doses		Three doses	
	18 years and older	93.7% (91.8 – 95.2)	95.7% (93.5 – 97.2)	--	--
	18–44 years	97.5% (96.1 – 98.3)	97.9% (94.3 – 99.2)	--	--
	45–59 years	91.6% (86.0 – 94.9)	93.5% (86.3 – 96.9)	--	--
	60–69 years	94.4% (87.6 – 97.5)	97.8% (91.0 – 99.4)	--	--
	70–79 years	93.7% (86.0 – 97.2)	95.5% (88.1 – 98.3)	--	--
	80 years and older	89.7% (77.0 – 95.4)	92.3% (81.4 – 96.8)	--	--
Heterologous Booster					
Combination: BNT162b2 + BNT162b2 + mRNA-1273 (n 527 225)					
	18 years and older	· ·	· ·	87.5% (83.6–90.4)	92.2% (87.7–95.1)
	18–44 years	· ·	· ·	85.9% (70.7–93.2)	--
	45–59 years	· ·	· ·	78.3% (62.5–87.4)	79.5% (48.1–91.9)
	60–69 years	· ·	· ·	86.6% (78.5–91.6)	93.5% (84.0–97.3)
	70–79 years	· ·	· ·	90.1% (82.1–94.6)	90.1% (80.0–95.1)
	80 years and older	· ·	· ·	91.3% (65.3–97.8)	95.2% (65.5–99.3)
Combination: BNT162b2 + BNT162b2 + ChAdOx1 nCoV-19 (n 477 208)					
	18 years and older	· ·	· ·	85.4% (80.8–88.8)	91.2% (85.9–94.5)
	18–44 years	· ·	· ·	76.3% (52.4–88.1)	--
	45–59 years	· ·	· ·	57.7% (35.8–72.1)	84.3% (55.9–94.4)
	60–69 years	· ·	· ·	91.8% (84.1–95.8)	82.9% (67.3–91.0)
	70–79 years	· ·	· ·	90.2% (80.3–95.1)	93.1% (81.5–97.4)
	80 years and older	· ·	· ·	--	--
Combination: CoronaVac + CoronaVac + BNT162b2 (442 836)					
	18 years and older	· ·	· ·	85.9% (82.8–88.5)	87.1% (83.7–89.8)
	18–44 years	· ·	· ·	93.0% (71.5–98.3)	--
	45–59 years	· ·	· ·	73.0% (26.6–90.0)	--
	60–69 years	· ·	· ·	92.1% (68.3–98.0)	86.0% (43.6–96.5)
	70–79 years	· ·	· ·	91.5% (85.8–94.9)	94.3% (88.0–97.3)
	80 years and older	· ·	· ·	83.5% (79.1–86.9)	84.8% (80.3–88.3)
Combination: CoronaVac + CoronaVac + ChAdOx1 nCoV-19 (280 245)					
	18 years and older	· ·	· ·	82.8% (78.4–86.3)	83.6% (78.7–87.5)
	18–44 years	· ·	· ·	87.5% (60.2–96.1)	--
	45–59 years	· ·	· ·	79.6% (44.5–92.5)	86.9% (52.0–98.2)
	60–69 years	· ·	· ·	80.6% (62.2–90.0)	89.2% (66.1–96.6)
	70–79 years	· ·	· ·	85.6% (77.3–90.9)	83.3% (72.8–89.7)
	80 years and older	· ·	· ·	81.8% (75.0–86.7)	81.9% (74.7–87.1)
Combination: CoronaVac + CoronaVac + mRNA-1273 (313 044)					
	18 years and older	· ·	· ·	85.5% (82.5–87.9)	87.8% (84.8–90.1)
	18–44 years	· ·	· ·	94.5% (60.2–99.2)	-
	45–59 years	· ·	· ·	64.1% (22.6–83.3)	--
	60–69 years	· ·	· ·	75.9% (55.7–86.8)	78.0% (50.1–90.3)
	70–79 years	· ·	· ·	88.6% (83.6–92.1)	91.1% (85.8–94.4)
	80 years and older	· ·	· ·	86.0% (82.2–89.0)	87.3% (83.5–90.2)

.. Not applicable -- Not estimable

The aVE for CS + booster was 80.2% (78.7–81.6). In this case, the highest effectiveness in preventing hospitalization occurred among those aged 18 to 44 years, followed by 70 to 79 years, with significant differences with respect to the other age groups. Furthermore, the highest efficacies were obtained for those who received three doses of BNT162b2 and for those with two doses of Ad26.COV2-S. It is worth noting that while three doses of CoronaVac had the lowest aVE compared to the other combinations studied, the effectiveness in preventing hospitalization due to COVID-19 increased when those who had two doses of CoronaVac received a heterologous booster (BNT162b2, mRNA-1273 or ChAdOx1 nCoV-19), and the differences disappeared.

### Effectiveness in preventing death

For the CS, the adjusted effectiveness in preventing death was 86.0% (95%CI 85.5–86.5) among adults aged 18 years and older. This aVE was highest for the youngest age group (18 to 44 years) and tended to decrease with age, such that the effectiveness for adults aged 80 years and older was 17.5% lower, with significant differences. For the CS, mRNA-1273 and BNT162b2 presented the highest effectiveness and CoronaVac again presented the lowest (14.4% lower than mRNA-1273), with significant differences. Statistical differences were also identified among the platforms, in that the heterologous CS was 13.7% more effective in preventing death from COVID-19 than the homologous series.

**Fig 7:**
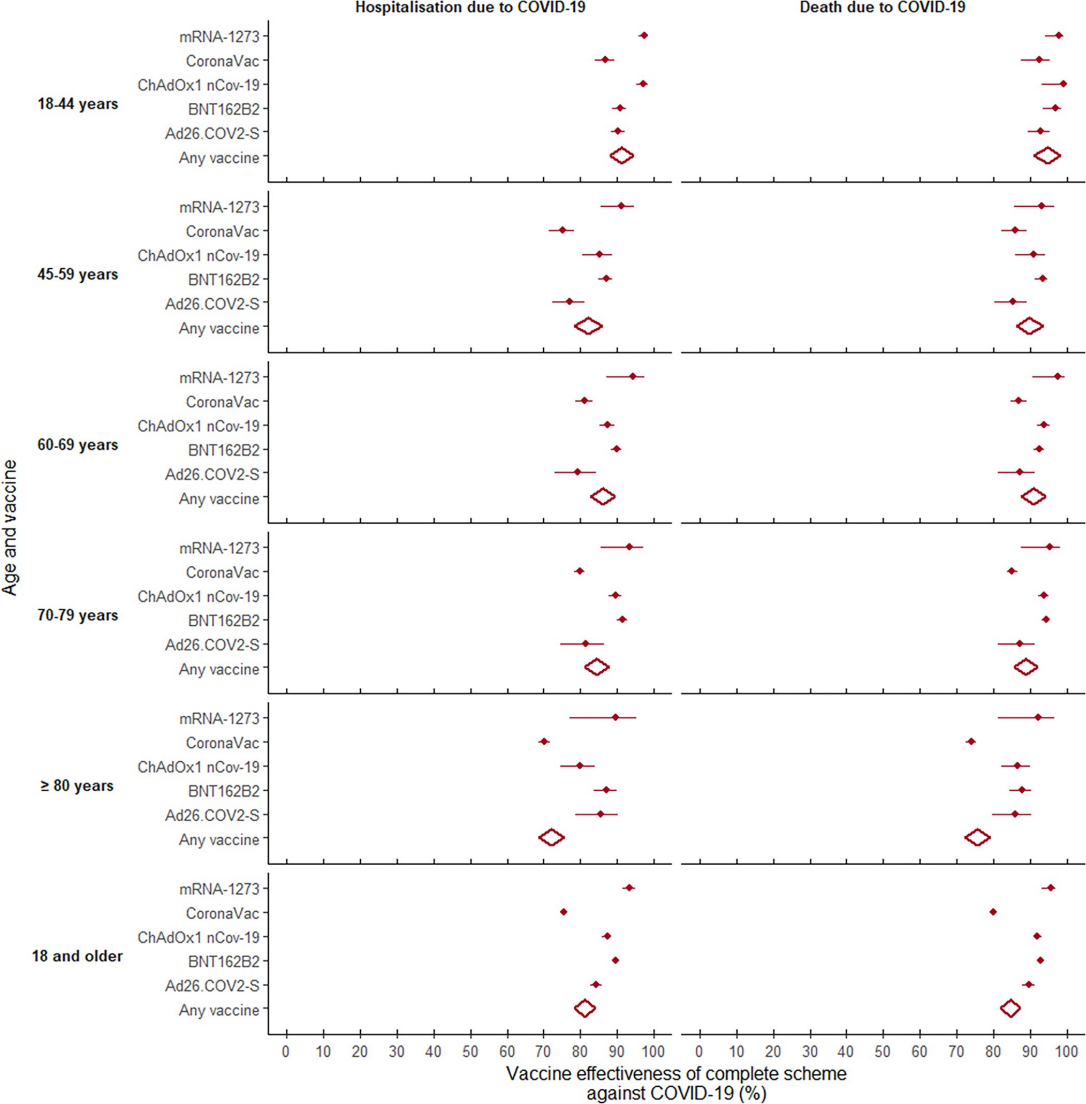
Forest plot of vaccine effectiveness in preventing hospitalization and death from COVID-19 among adults aged 18 years and older in Colombia, by age group and vaccine: Complete series.

**Fig 8:**
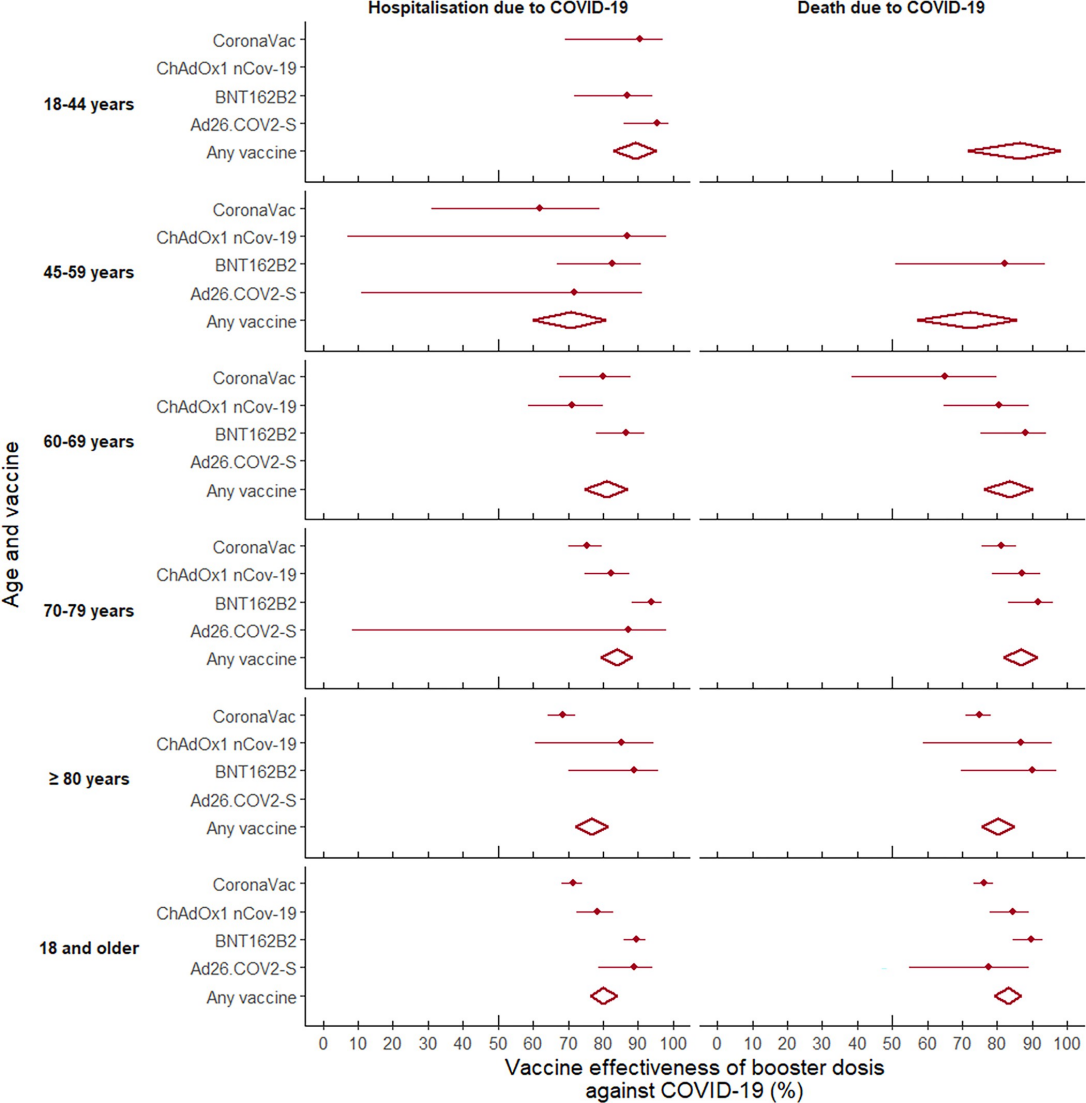
Forest plot of vaccine effectiveness in preventing hospitalization and death from COVID-19 among adults aged 18 years and older in Colombia, by age group and vaccine 2B: Complete series + booster.

With regard to CS + booster, this presented an aVE in preventing death of 83.1% (95%CI 81.5–84.5). In this case, no differences were found according to age, and although the heterologous booster was more effective, this was not statistically different from the homologous booster. The highest effectiveness in preventing death occurred among those who received two doses of BNT162b2 and a booster with mRNA-1273, with significant differences only compared to those who received two doses of CoronaVac and a booster of ChAdOx1 nCoV-19 or three doses of CoronaVac (Figs 7 and 8).

All estimators were significant (p<0.0001). The results were obtained from Cox proportional hazards survival models, adjusted for: age; sex; enrollment in the Colombian health system; diagnosis of cancer, diabetes, hypertension and chronic kidney disease; and municipality of residence. In all cases, the reference group corresponded to those who had not received any dose of the COVID-19 vaccine.

Finally, as part of the sensitivity analysis, the overall effectiveness, and by age group were estimated, before and after the emergence of the Omicron variable in Colombia (S2 Table). In addition, effectiveness was estimated for two different follow-up periods: 15–180 days after the application of the complete series or after the booster (S3 Table), and 181–360 days after the application of the complete series or booster (S4 Table). In all cases, although there are slight changes in effectiveness, the conclusions about effectiveness hold, as do observed differences in effectiveness between age groups.

## Discussion

This study evaluated the effectiveness of Ad26.COV2-S, BNT162b2, ChAdOx1 nCoV-19, CoronaVac and mRNA-1273 vaccines in preventing hospitalization and death among the Colombian population aged 18 years and older. It provides strong, real-world evidence that supports continuing and expanding COVID-19 vaccination, and particularly reinforces the need to increase the administration of boosters to prevent severe cases of the disease and to maintain vaccine protection over time.

Our analysis was based on a large sample size and a diversified portfolio existed in the country studied, the present study is one of the few population-based cohorts [22], conducted in low- and middle-income countries that enables conducting an age-stratified evaluation of the effectiveness of each series, with and without booster. In summary, while the effectiveness of vaccination was high for all the age groups and series analyzed, the detailed analysis provided herein makes it possible to distinguish and highlight relevant differences among the series and age groups, as we ourselves had previously described even within subgroups of older adults in Colombia [17].

The relevant findings of the present study is that the overall effectiveness of COVID-19 vaccination in adults aged 18 years and older in Colombia, after controlling for the main confounding variables, was over 80% in preventing both hospitalization and death due to SARS-CoV-2 infection, both for the CS and CS + booster, and as previously stated with differences among age groups and the vaccines received.

In particular, this analysis found that the effectiveness was higher for people who received heterologous vaccination series and for those who received RNA (mRNA) vaccines, while the effectiveness was significantly lower for people vaccinated with CoronaVac. Nevertheless, the effectiveness of this biologic was still high (at least 62.0% in this study) considering the standards initially established by the World Health Organization for COVID-19 vaccines [23], and especially from a public health perspective given the initial scarcity, that is, these values represent a large number of lives saved at the population level. The relative lower effectiveness of CoronaVac found by this study has been reported previously, and is due to the lower initial potency of the immune response [24, 25] as well as a more rapid loss of protection compared to the other vaccines analyzed, as measured by antibodies [26].

This study determined that the RNA (mRNA) vaccines performed better in preventing hospitalization and death among those with a CS, which is consistent with previous immunogenicity studies that found the neutralizing response to be better with RNA vaccines, with some variability according to age. Analytical observational studies have found the BNT162b2 and mRNA-1273 vaccines to be more effective than CoronaVac in preventing infection, as pointed out in the recent systematic meta-analysis by Zheng C et al. [15].

In the case of the complete series, this study found that the Moderna vaccine tended to be more effective in preventing hospitalization and death. Nonetheless, since this was the last biologic to become available in Colombia, there is currently insufficient information to estimate the effectiveness of the booster for those who received Moderna as their primary vaccination series.

These results are also similar to those reported by the randomized phase III clinical trials required to obtain emergency use authorizations, which estimated the efficacy of the vaccines in preventing laboratory-confirmed infection to be 66.9% (95%CI 59.0–73.4%) for Ad26.COV2.S (4), 91. 3% (95% CI 89.0% - 93.2%) for BNT162b2 (5), 74.0% (95% CI 65.3% - 80.5%) for ChAdOx1 nCoV-19 (6), 83.5% (95% CI 65.4% - 92.1%) for CoronaVac (7), and 94.1% (95% CI 89.3% - 96.8%) for mRNA-1273 [8], although these early studies could not determine the existence of significant differences among vaccines.

In addition, given the needs of the global situation, those clinical trials were conducted with a relatively short follow-up period, and therefore, effectiveness for outcomes that take longer to occur (such as hospitalization and death) could not be analyzed. They also could not predict the performance of the vaccines against emerging variants of the virus that were subsequently predominant. Thus, as this and other studies have found, in many cases the actual effectiveness of the vaccines was expected to be lower than initial estimates, especially in light of new scenarios involving epidemiological complexities nearly two years after vaccination programs began worldwide. However, population-based, observational studies such as this one, that have analyzed data that is routinely collected (administrative records) as national vaccination programs have been implemented, provide robust evidence of effectiveness and also make it possible to empirically evaluate the external validity of the results obtained with RCTs.

Regarding differences by age group, the estimated effectiveness of all the vaccines studied tended to be higher among young and middle-aged adults and relatively lower among older adults, which has also been shown by previous research in the United States [27, 28], Qatar [29], Brazil [30] and Israel [31].

In this analysis, the overall effectiveness for adults aged 80 years and older ranged from 75.2% (for preventing hospitalization with CS) to 80.3% (for preventing death with CS + booster). Although these were high for preventing the relevant outcomes in this age group, they were significantly lower compared to younger groups. This finding is also consistent with observational studies conducted with similar populations in Chile and Brazil that included the CoronaVac platform [32], as well as with the international literature [33, 34]. This could be explained by a higher prevalence of multimorbidity in the elderly, which is associated with frailty from the aging process [35] as well as immunosenescence [36], which can lower the immune response and reduce the protection that is provided by vaccination. All of the above serve as a reminder of the importance of considering the role of age in the natural and vaccine-induced immune response, as well as the need to more vigorously increase the administration of booster doses among older adults in current and future COVID-19 vaccination programs [22].

It is noteworthy that according to the findings herein, there do not appear to be large significant differences in protection between the complete series and the complete series + booster. It should be noted that in the vast majority of cases the confidence intervals are very wide and overlap each other, which may be due to the reduced statistical power to compare the two groups because this investigation stratified by age and vaccine, as well as the substantial reduction in mortality and hospitalization that started to occur before boosters began to be administered in the country in the middle of 2022.

In addition, a difference might have been found if it were possible to exclusively make a comparison to subjects who had finished the complete series much earlier and had not received a booster, which was not possible with this matched design. It is also possible that the difference is only evident with longer observation periods, as in studies conducted in high-income countries. Therefore, this exercise needs to be replicated when more time has passed, after a larger proportion of people in the country receives the booster.

Additionally, considering that Colombia had a high attack rate of infection, the possibility that people with a complete series could have been naturally exposed to the virus at that point in the pandemic should be considered, which at that point would have provided additional protection as a result of hybrid immunity, making them comparable to receiving the booster. This situation could explain why the differences in protection between those vaccinated with a CS and those who also received the booster were not so evident in countries such as Colombia, since all of them had high exposure to the virus. It is also possible that those less likely to receive the booster were the most likely to be infected, although information is only available on confirmed infection, and with the reduction in the severity of symptoms over time fewer infected people would have sought medical care, and therefore, would not have been detected. That is, people who only received the complete series versus those who also received boosters may differ in terms of observable and unobservable characteristics that can explain differences in risk. Unfortunately, the present study cannot evaluate these hypotheses. A comparison would involve more complex designs that allow for adjusting for the calendar period and for the dominant variant at each point in time to evaluate effectiveness by period, which was not possible in this study because this information is not available in the country.

Another relevant limitation of this study was that it was not possible to evaluate effectiveness according to the dominant variants that emerged over time. While in Colombia it is not possible to determine the variants involved in each case, the fact that effectiveness continued over time may constitute indirect evidence that the vaccines studied were effective in the case of new variants, at least in preventing severe cases and death. In this regard, Link-Gelles et al. [37] reported a decrease in the effectiveness of the vaccines during the circulation of Omicron sublines in the United States, which is why they have recommended that adults receive two booster doses. In Colombia, the Omicron sublineages (BA.1.X, BA.2.X, BA.2.12.1 and BA.4) were predominant from December 2021 until the end of the observation period [38], and although this study could not identify decreases in immunity over time or changes in vaccine effectiveness according to the predominant variant, the recommendation should be that all adults stay current with their COVID-19 vaccinations, including boosters.

However, as an approximation to assess whether the changes in overall effectiveness, and between age groups, could be explained by changes over time in the predominant variant in the country (especially considering that the subjects were vaccinated at different times given the stages by age of the National Vaccination Plan), we carried out a sensitivity analysis that stratified by two calendar periods, and by two follow-up periods. Although our results confirmed that, the effectiveness by age is very similar between both calendar periods, and between both follow-up periods. In the latter case, although the effectiveness certainly decreases over time, the differences in effectiveness observed by age group remain.

Our approach is exploratory and to better assess these differences in effectiveness by variant and their changes over time, other study designs with different methodological approaches would be required. Determining effectiveness over calendar time and its change over time since vaccination (two aspects methodologically difficult to separate) is a major methodological challenge in effectiveness studies and is likely to require other, more sophisticated methodological approaches, and ideally at from cohort studies using primary sources data. It is important to consider that in certain low-risk groups, the stratification to a certain period produces unstable estimates, due to the low occurrence, especially when the vaccinated began to receive boosters. As already mentioned, other limitations of this analysis have to do with the fact that, despite the large sample size, both the denominator and the number of events were significantly smaller in the more specific analyses, affecting the statistical power of the estimates. Thus, these results are more accurate for the CS analysis than for the boosters because the latter involved less person-time and events. In fact, in some cases it was not possible to estimate the effectiveness of CS + booster due to the reduced person-time.

It is generally a limitation of the study that the protective effects of vaccines may be mixed with the effects of natural exposure to the virus among both cohorts. Certainly, in both study groups (vaccinated and unvaccinated), there were individuals who may have been infected at some point. Although the stratification we performed by COVID-19 history partially addresses this issue, it depends on access to effective diagnostic tests, which can also confound the results. However, it is important to consider that our study accounted for locality and time, as components of the matching process. Given that pandemic peaks occur at specific points in space/time, it is reasonable to assume that, on average, vaccinated and unvaccinated subjects at each point in time in the same location were exposed to the same incidence. Therefore, the probability of being infected, given also the individual covariate adjustments, is expected to be partially controlled due to the study design. We have represented these complex relationships comprehensively in S3 Fig, which guided our analysis.

However, this approach is imperfect, and certainly, the estimated effectiveness combines the effects of natural immunity (including undetected infections) with the effects of vaccines. Separating these effects would require more sophisticated statistical approaches and methods beyond the scope of this study. Nevertheless, we believe that our results provide a good estimation of the average effectiveness over the study period.

In addition, as with any analytical, observational study, this work may present some degree of residual confounding. In this case, it is possible that people who had not received any dose of the COVID-19 vaccine in Colombia were different from those who had been vaccinated in terms of both access to vaccination and the negative outcomes of interest (hospitalization and death). It is even possible that those who received the booster also differ in these ways from those who received only the complete series. Therefore, the statistical model was adjusted for possible confounding demographic, socioeconomic and clinical variables, such as the presence of underlying diseases that could confound or alter the magnitude of the estimates (see DAG in S3 Fig). However, this estimation process is subject to the assumption that all relevant confounding variables were considered and that the measurement error of the confounders was small.

Additionally, a limitation of our study arises from defining the unvaccinated group as those who remained unvaccinated throughout the entire study period. This essentially conditions on a future known event—not receiving a vaccination at any point during the study—which may diverge from the ideal assumptions of a clinical trial. We partially mitigated this limitation by adjusting for major sources of heterogeneity between the vaccinated and unvaccinated groups. However, if additional factors not only associate with being unvaccinated but also with persistently remaining unvaccinated, residual confounding that could influence the estimates may exist. Nevertheless, our primary aim is not to mirror efficacy under controlled conditions, but to estimate real-world effectiveness to inform public health policy. In this context, using as a reference those individuals who remained unvaccinated throughout the entire study period is highly relevant.

Similarly, this challenge can arise when estimating the effectiveness of boosters compared to the unvaccinated, as the unvaccinated group throughout the follow-up represents an oversampled population that may be heterogeneous compared to those who received at least one vaccine. This could be another potential source of residual confusion. Nevertheless, for the purpose of informing public policy, we consider it valuable to estimate the effectiveness of the booster during the study period in relation to remaining unvaccinated.

Another limitation of this study, like many analyzes of effectiveness in real-world conditions, is that it is impossible for the surveillance system to detect all cases of SARS-CoV-2 infection. However, this would have few effects on the estimators of the effectiveness at hospitalization and at death, where they depend on the clinical outcome, and less on access to tests, especially considering that in Colombia, access to tests in the hospital setting was relatively good.

Despite their limitations, population-based cohort studies are one of the best approaches for providing evidence of real-world effectiveness. The broad portfolio of vaccines contained in Colombia’s NVP plus the inclusion of over 14 million individuals in the analysis made it possible to evaluate vaccine effectiveness in a middle-income Latin American country. Thus, this study provides robust evidence on the performance of different vaccines in an uncontrolled, real-world setting, and during a period when Mu (B.1 .621), Delta (B.1.617) and Omicron (BA.1.X, BA.2.X, BA.2.12.1, BA.4) were predominant (38). This evidence may also be useful in other similar contexts, such as low- and middle-income countries that also administered these vaccines, and especially those with diversified series and boosters.

It is also worth noting that several authors have published analyses on the effectiveness of vaccines in real-world conditions. Nonetheless, some of those studies used short follow-up periods [32, 39–42], only analyzed the effectiveness of partial vaccination [43], were specific to the population groups of interest [43] or analyzed a single vaccine [32, 39, 44] or a single vaccine platform [40, 42]. Few studies have been conducted like the one herein, with: 1) the analysis of the effectiveness of a complete series and a complete series plus booster for critical outcomes such as death from COVID-19, 2) a sample of adults aged 18 years and older, 3) the evaluation of five vaccines, 4) the assessment of different platforms, 5) a study period of over one year and 6) the inclusion of more than 14 million individuals.

Furthermore, our results indirectly show a partial reduction in the inequities in the distribution of COVID-19 vaccines worldwide, thanks to the sound decisions that were made by some middle-income countries such as Colombia, which constructed diversified vaccine protocols and prioritized populations to manage the initial shortage [19]. As a result, this study shows that some of the combinations that include platforms such as an inactivated virus (CoronaVac) are effective, especially when accompanied by subsequent heterologous boosters, even though separately they are less effective than RNA (mRNA), as several head-to-head evaluations have shown [45].

During the conceptualization and design of the vaccination portfolios, low- and middle-income countries such as Colombia had to exert a great deal of effort to obtain vaccines due to competition with hoarding by high-income countries. Under conditions of uncertainty and strong political pressure, acquiring the vaccines that were available to build a diversified portfolio resulted in saving more lives than if they had waited for vaccines with higher effectiveness, especially when initially there was no knowledge of the actual differences among the vaccines.

## Conclusion

COVID-19 vaccination is currently being promoted among the general population. The results herein reinforce the relevance of the recommendation and offer the hope that the pandemic can be controlled in the near future through mass vaccination. The Esperanza cohort was so named because hope, which together with knowledge, is what mobilized collective solidarity, and today brings us close to overcoming this threat to life.

However, although this study presents valuable insights into the real-world effectiveness of vaccination against COVID-19, it is essential to acknowledge the inherent limitations in accurately disentangling waning immunity and the distinct contributions of different Variants of Concern (VoC) to the estimated results. Furthermore, it should be considered that this type of design has several inherent limitations, such as the difficulty in evaluating ecological and indirect effects of protection, which may arise from the reduction of transmission leading to reduced exposure among high-risk individuals and subsequent mortality reduction. This complexity is compounded by factors such as increased natural exposure, the emergence of hybrid immunity, and the aforementioned variants, presenting a methodological challenge for real-world effectiveness studies.

## Supporting information

S1 Fig Flowchart: Assembly of the database for the present analysis. (TIF)

S2 Fig Verification of proportional hazards assumptions. (TIF)

S3 Fig Directed acyclic graph considered for the selection of confounding variables in the regression models. (TIF)

S1 Table Cut-off points for the chronological sensitivity analysis according dominant variant in Colombia, 2021–2022. (DOCX)

S2 Table Sensitivity analysis according to calendar time. Effectiveness of COVID-19 vaccines in preventing hospitalization and death due to COVID-19 among adults 18 years and older by age group. February–November 2021 vs December 2021 –June 2022.
(DOCX)

S3 Table Sensitivity analysis according to time of exposure to the vaccine. Effectiveness of COVID-19 vaccines in preventing hospitalization and death in adults 18 years and older by age group. 15–180 days after the application of the complete series or after the booster.
(DOCX)

S4 Table Sensitivity analysis according to time of exposure to the vaccine. Effectiveness of COVID-19 vaccines in preventing hospitalization and death in adults 18 years and older by age group. 181–360 days after the application of the complete series or after the booster.
(DOCX)
