# Supplementary figures and images for: Real-world effectiveness of COVID-19 vaccines among Colombian adults: A retrospective, population-based study of the ESPERANZA cohort
Source: PLOS Glob Public Health. 2023 Sep 8;3(9):e0001845. doi: 10.1371/journal.pgph.0001845 (PMC10491003; doi:10.1371/journal.pgph.0001845)

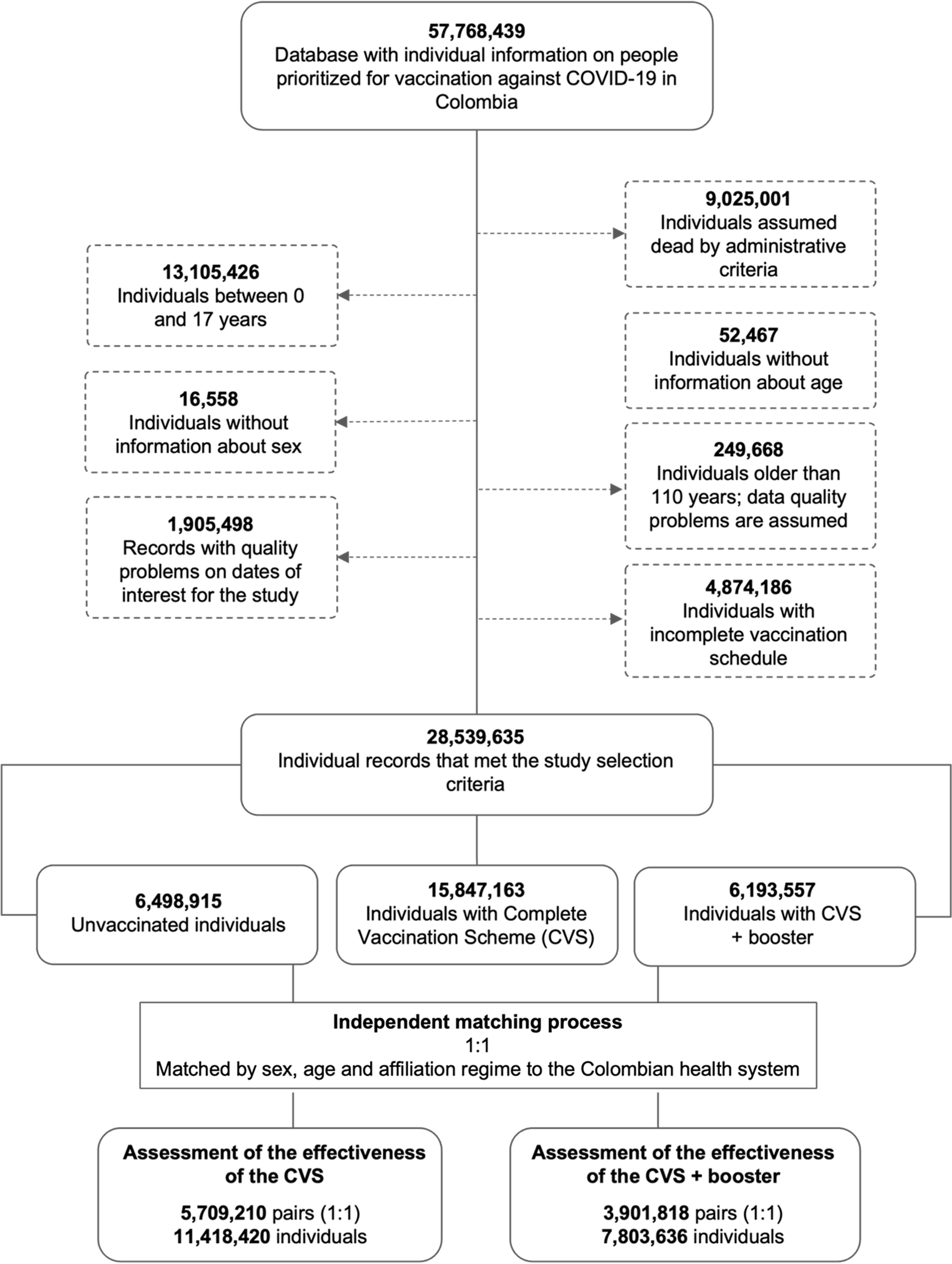

Supplement: S1 Fig — (TIF) [file pgph.0001845.s001.tif]

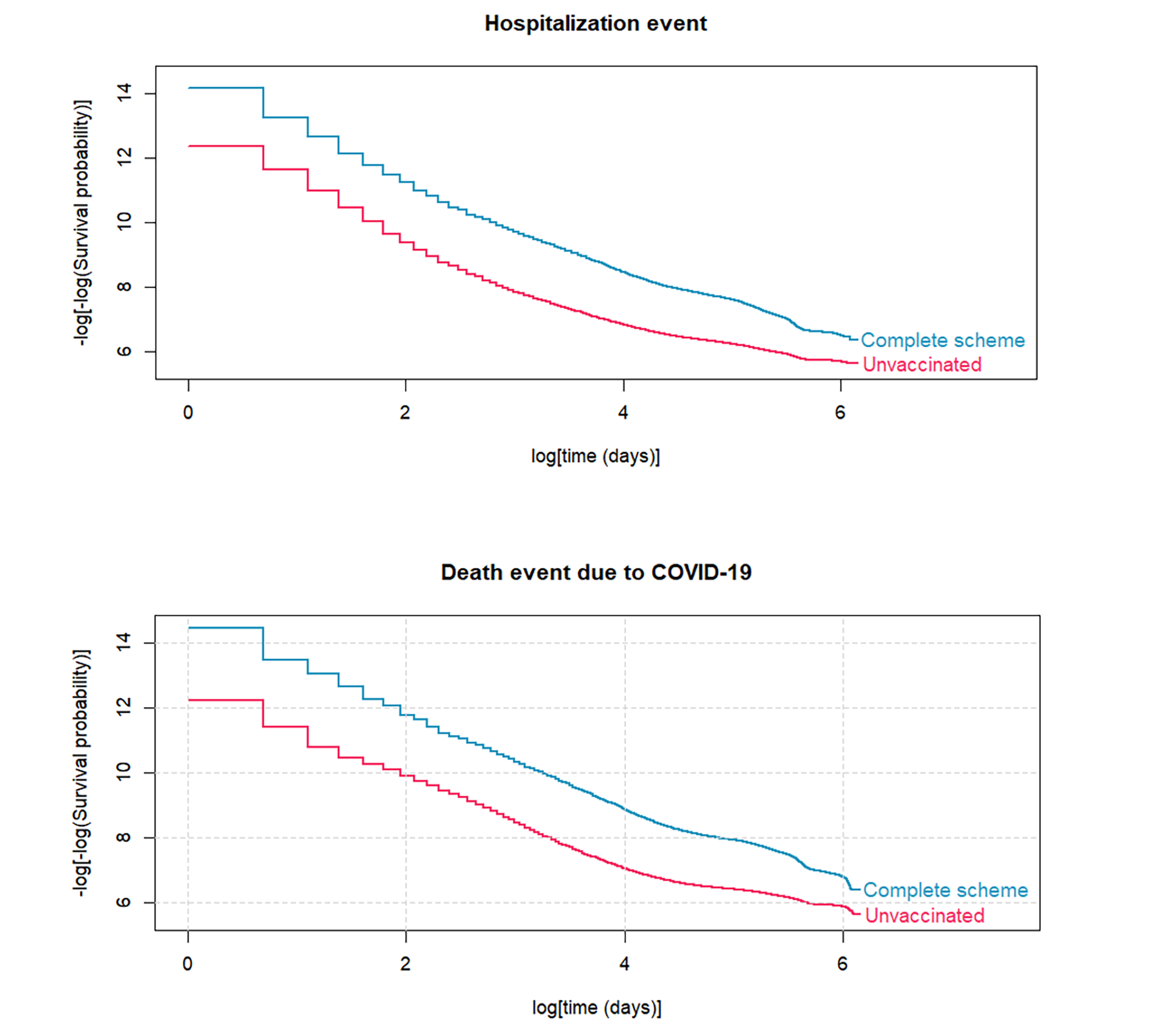

Supplement: S2 Fig — (TIF) [file pgph.0001845.s002.tif]

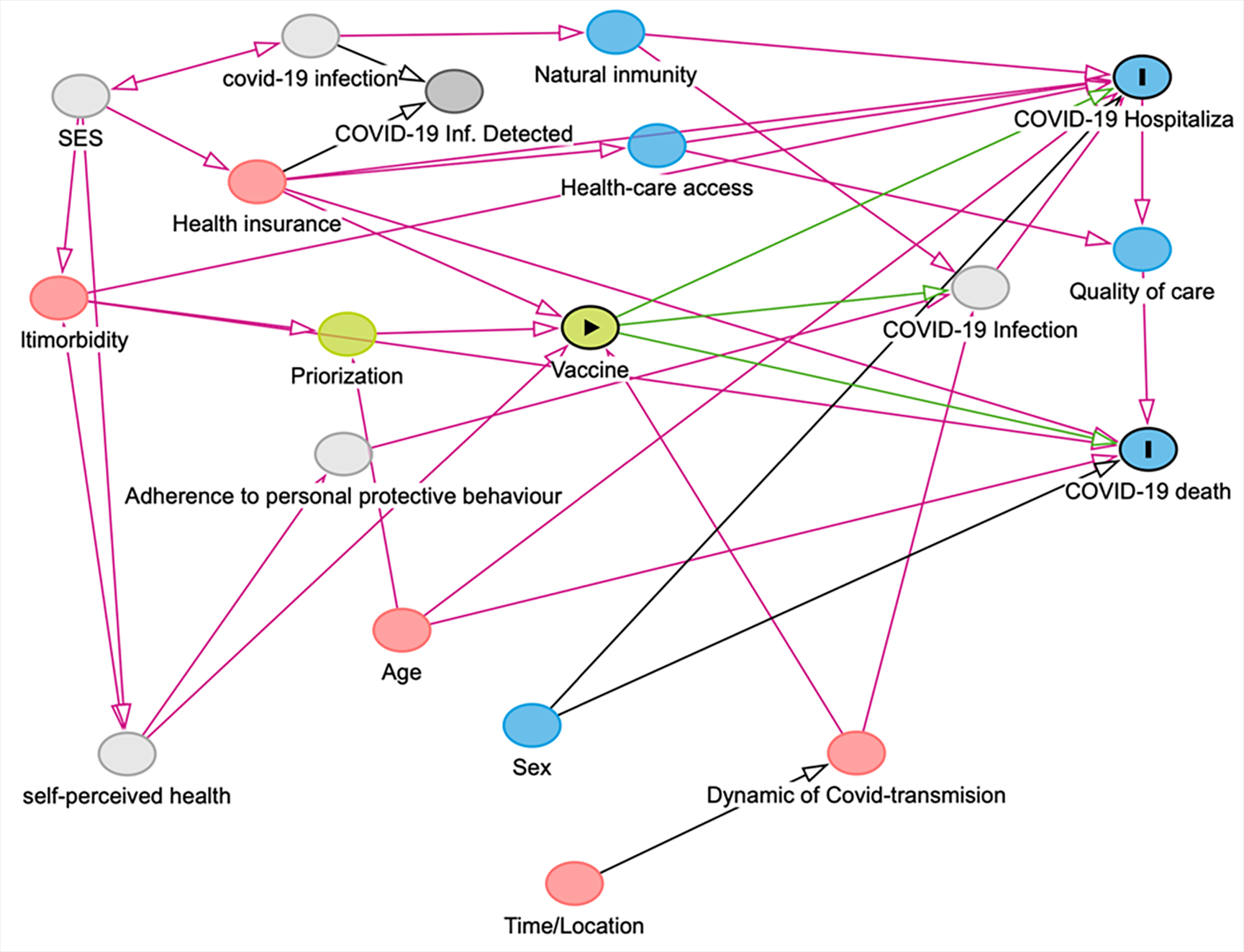

Supplement: S3 Fig — (TIF) [file pgph.0001845.s003.tif]
