# Supplementary material for: Real-world effectiveness of COVID-19 vaccines among Colombian adults: A retrospective, population-based study of the ESPERANZA cohort
Source: PLOS Glob Public Health. 2023 Sep 8;3(9):e0001845. doi: 10.1371/journal.pgph.0001845 (PMC10491003; doi:10.1371/journal.pgph.0001845)
Supplement: S1 Table — (DOCX) [file pgph.0001845.s004.docx]

**S1 Table. Cut-off points for the chronological sensitivity analysis according to dominant variant in Colombia, 2021-2022.**

| **Dates** | **Dominant variant** |
| --- | --- |
| February – August 2021 | Mu |
| September – November 2021 | Delta |
| December 2021 – March 2022 | Omicron |
| April – May 2022 | BA.2.x ( Omicron Sublineage ) |
| June – July 2022 | BA.4 ( Omicron Sublineage ) |

Source: Colombian National Health Institute.

Original data available in: <https://www.ins.gov.co/Noticias/Paginas/coronavirus-genoma.aspx>
