# Supplementary material for: Real-world effectiveness of COVID-19 vaccines among Colombian adults: A retrospective, population-based study of the ESPERANZA cohort
Source: PLOS Glob Public Health. 2023 Sep 8;3(9):e0001845. doi: 10.1371/journal.pgph.0001845 (PMC10491003; doi:10.1371/journal.pgph.0001845)
Supplement: S2 Table — Effectiveness of COVID-19 vaccines in preventing hospitalization and death due to COVID-19 among adults 18 years and older by age group. February–November 2021 vs December 2021 –June 2022. (DOCX) [file pgph.0001845.s005.docx]

**S2 Table. Sensitivity analysis according to calendar time. Effectiveness of COVID-19 vaccines in preventing hospitalization and death due to COVID-19 among adults 18 years and older by age group. February – November 2021 vs December 2021 – June 2022**

|  | **Complete series**  **(95% CI)** | | **Complete series + booster**  **(95% CI)** | |
| --- | --- | --- | --- | --- |
|  | **Hospitalization** | **Death** | **Hospitalization** | **Death** |
| **February – November 2021 (Dominant variant: Mu/Delta)** | | | | |
| Age group |  |  |  |  |
| **18 years and over** | **74.3 (73.5 – 75.1)** | **79.9 (79.1 – 80.6)** | *--* | *--* |
| 18 – 44 years | 80.1 (78.0 – 81.9) | 90.0 (87.2 – 92.2) | *--* | *--* |
| 45 – 59 years | 74.6 (72.3 – 76.8) | 83.3 (80.7 – 85.6) | *--* | *--* |
| 60 – 69 years | 80.4 (78.7 – 81.9) | 85.1 (83.4 – 86.7) | *--* | *--* |
| 70 – 79 years | 79.5 (78.1 – 80.9) | 85.5 (84.2 – 86.6) | *--* | *--* |
| 80 years and over | 56.0 (53.7 – 58.2) | 68.0 (66.3 – 69.5) | *--* | *--* |
| **December 2021 – June 2022 (Dominant variant: Omicron)** | | | | |
| Age group |  |  |  |  |
| **18 years and over** | **85.4 (82.2 – 88.0)** | **60.7 (47.1 – 70.8)** | **76.6 (73.7 – 79.2)** | **78.6 (75.2 – 81.6)** |
| 18 – 44 years | 96.6 (95.5 – 97.5) | 94.0 (88.3 – 97.0) | 91.2 (86.3 – 94.3) | 90.4 (76.0 – 96.2) |
| 45 – 59 years | *--* | *--* | 74.8 (65.1 – 81.7) | 72.6 (53.5 – 83.8) |
| 60 – 69 years | *--* | -- | 77.1 (70.3 – 82.3) | 83.6 (76.5 – 88.6) |
| 70 – 79 years | 28.6 (0.0 – 59.5) | 39.8 (8.5 – 66.6) | 83.1 (79.1 – 86.4) | 86.3 (82.0 – 89.6) |
| 80 years and over | 51.2 (2.3 – 75.6) | 20.4 (0.0 – 61.8) | 58.2 (47.8 – 66.5) | 62.2 (51.9 – 70.2) |

*· · Not applicable -- Not estimable.*

All estimators were significant (p<0.0001). The results were obtained from Cox proportional hazards survival models, adjusted for age, sex, affiliation regime to the Colombian health system, diagnosis of cancer, diabetes, hypertension, chronic kidney disease, history of COVID-19 infection and residence municipality. In all cases, the reference group corresponds to people who have not received any dose of any COVID-19 vaccine. Note: The analysis for February – November used the same approach applied for the entire period with censorship as of November 30. On the other hand, the analysis of December 2021 – June 2022 included only those who did not present hospitalization or death before November, 2021, including vaccinated and unvaccinated who survived both events during the first period (February – November 2021).
