# Supplementary material for: Real-world effectiveness of COVID-19 vaccines among Colombian adults: A retrospective, population-based study of the ESPERANZA cohort
Source: PLOS Glob Public Health. 2023 Sep 8;3(9):e0001845. doi: 10.1371/journal.pgph.0001845 (PMC10491003; doi:10.1371/journal.pgph.0001845)
Supplement: S3 Table — Effectiveness of COVID-19 vaccines in preventing hospitalization and death in adults 18 years and older by age group. 15–180 days after the application of the complete series or after the booster. (DOCX) [file pgph.0001845.s006.docx]

**S3 Table. Sensitivity analysis according to time of exposure to the vaccine. Effectiveness of COVID-19 vaccines in preventing hospitalization and death in adults 18 years and older by age group. 15 – 180 days after the application of the complete series or after the booster**

|  | **Complete series**  **(95% CI)** | | **Complete series + booster**  **(95% CI)** | |
| --- | --- | --- | --- | --- |
|  | **Hospitalization** | **Death** | **Hospitalization** | **Death** |
| Age group |  |  |  |  |
| **18 years and over** | **85.6 (85.1 – 86.2)** | **88.7 (88.2 – 89.2)** | **80.3 (78.8 – 81.7)** | **83.6 (82.0 – 85.0)** |
| 18 – 44 years | 92.5 (91.4 – 93.4) | 96.1 (94.6 – 97.2) | 88.7 (83.5 – 92.3) | 89.7 (74.0 – 95.9) |
| 45 – 59 years | 85.6 (84.0 – 87.1) | 92.2 (90.7 – 93.5) | 70.2 (60.7 – 77.5) | 73.9 (59.5 – 83.3) |
| 60 – 69 years | 89.7 (88.6 – 90.8) | 94.3 (93.4 – 95.1) | 81.8 (77.5 – 85.2) | 85.1 (8.0 – 88.9) |
| 70 – 79 years | 88.2 (87.2 – 89.2) | 91.6 (90.7 – 92.4) | 84.2 (81.8 – 86.4) | 87.2 (84.6 – 89.4) |
| 80 years and over | 78.4 (77.0 – 79.7) | 81.5 (80.3 – 82.6) | 77.1 (74.5 – 79.4) | 80.7 (78.2 – 82.9) |

*· · Not applicable -- Not estimable*

All estimators were significant (p<0.0001). The results were obtained from Cox proportional hazards survival models, adjusted for age, sex, affiliation regime to the Colombian health system, diagnosis of cancer, diabetes, hypertension and chronic kidney disease, and municipality of residence. In all cases, the reference group corresponds to people who have not received any dose of the COVID-19 vaccine.
